# Cytomegalovirus-Induced Hypertensive Anterior Uveitis: Diagnostic Challenge in an Immunocompetent Patient

**DOI:** 10.7759/cureus.52826

**Published:** 2024-01-23

**Authors:** João Romano, Gonçalo Godinho, João Chaves, Nuno Oliveira, João P Sousa

**Affiliations:** 1 Ophthalmology, Centro Hospitalar de Leiria, Leiria, PRT

**Keywords:** angle closure glaucoma, uveitic glaucoma, hypertensive anterior uveitis, posner-schlossman syndrome, intraocular pressure management, uveitis, glaucoma, immunocompetent, cytomegalovirus, anterior uveitis

## Abstract

Hypertensive anterior uveitis poses diagnostic challenges owing to its multiple potential etiologies. Cytomegalovirus (CMV) infection is an under-recognized cause that exhibits diverse clinical presentations. This case report focuses on the intricate diagnostic challenge encountered in a 66-year-old immunocompetent patient with CMV-induced hypertensive anterior uveitis. The patient's history, encompassing angle closure glaucoma and topiramate use, contributed to the hypertensive crisis. Initial management included intraocular pressure (IOP)-lowering medication, topiramate discontinuation, and bilateral phacoemulsification, successfully normalizing IOP. However, a subsequent recurrence prompted a detailed investigation. The identification of keratic precipitates and a synechial closed angle led to aqueous humor sampling and polymerase chain reaction (PCR) testing, unveiling the presence of CMV-DNA. Treatment led to a favorable response, resolving ocular inflammation and effectively controlling IOP. This case underscores the complexity of diagnosing and managing CMV-induced hypertensive anterior uveitis, emphasizing the critical role of a comprehensive approach in achieving successful outcomes.

## Introduction

Hypertensive anterior uveitis is a challenging and often confounding ocular condition that can pose a diagnostic dilemma. Among the various etiologies responsible for this rare form of uveitis, cytomegalovirus (CMV) infection stands out as a significant yet under-recognized cause [1]. Its clinical manifestations can vary, encompassing self-limiting iritis with sector iris atrophy, acute relapsing hypertensive anterior uveitis akin to Posner-Schlossman syndrome or a chronic anterior uveitis sharing features with Fuchs uveitis syndrome [2-5]. Distinguished by low-grade anterior segment inflammation and mild ocular symptoms without synechiae, CMV anterior uveitis stands apart from anterior uveitis caused by herpes simplex virus (HSV) or varicella-zoster virus (VZV) [1,3].

This case report aims to shed light on the complexities of diagnosing and managing CMV-induced hypertensive anterior uveitis in a 66-year-old immunocompetent patient.

## Case presentation

A 66-year-old female presented to our emergency department with complaints of pain and blurred vision in her left eye for two days, with no further ophthalmological or systemic complaints. Her past ocular history was significant for acute angle closure glaucoma, which had been treated with bilateral iridotomy two years prior. The patient's past medical history was significant for depression and migraine. Notably, she had been taking topiramate for one month before the onset of ocular symptoms for migraine prophylaxis.

Upon examination, the patient's visual acuity was 20/25 in the right eye but reduced to counting fingers in the left eye. The left pupil exhibited fixed mydriasis, and Goldmann applanation tonometry revealed a markedly elevated intraocular pressure (IOP) of 55 mmHg in that eye, while measuring 21 mmHg in the right eye. Slit-lamp examination demonstrated shallow anterior chambers and cataracts in both eyes, along with ciliary flush, corneal edema, and uncertainty regarding the patency of the previous iridotomy in the left eye. Fundoscopic examination revealed an optic disc with a cup-disc ratio of 0.2 in the right eye and 0.5 in the left eye, with the remainder being unremarkable.

Immediate management involved the administration of intravenous mannitol (2 g/kg IV infused over 60 minutes) and oral hyperosmotics (acetazolamide 500mg), resulting in a reduction of the left eye's IOP to 42 mmHg. A new laser peripheral iridotomy was not possible to perform because of corneal edema. The patient was prescribed topical (timolol 0.5% -dorzolamide 2% fixed combination twice a day and brimonidine 0.15% twice a day) and oral hypotensive medication (acetazolamide 250mg every eight hours), as well as topical corticosteroid (dexamethasone phosphate 0.1% every three hours). Furthermore, topiramate was discontinued. Over the subsequent days, the patient's intraocular pressure (IOP) stabilized at around 15 mmHg, and the patency of both iridotomies was confirmed. However, the anterior chambers remained shallow, and angles were closed on gonioscopy.

To address the shallow anterior chambers, the patient underwent bilateral uncomplicated phacoemulsification (monofocal intraocular lens +28.00D was implanted in the right eye and +26.00D in the left eye), which led to the normalization of intraocular pressure without the need for hypotensive medication. One month after surgery, her best-corrected visual acuity (BCVA) was 20/20 in the right eye and 20/60 in the left eye, and her IOP was 16 mmHg in both eyes.

**Figure 1:**
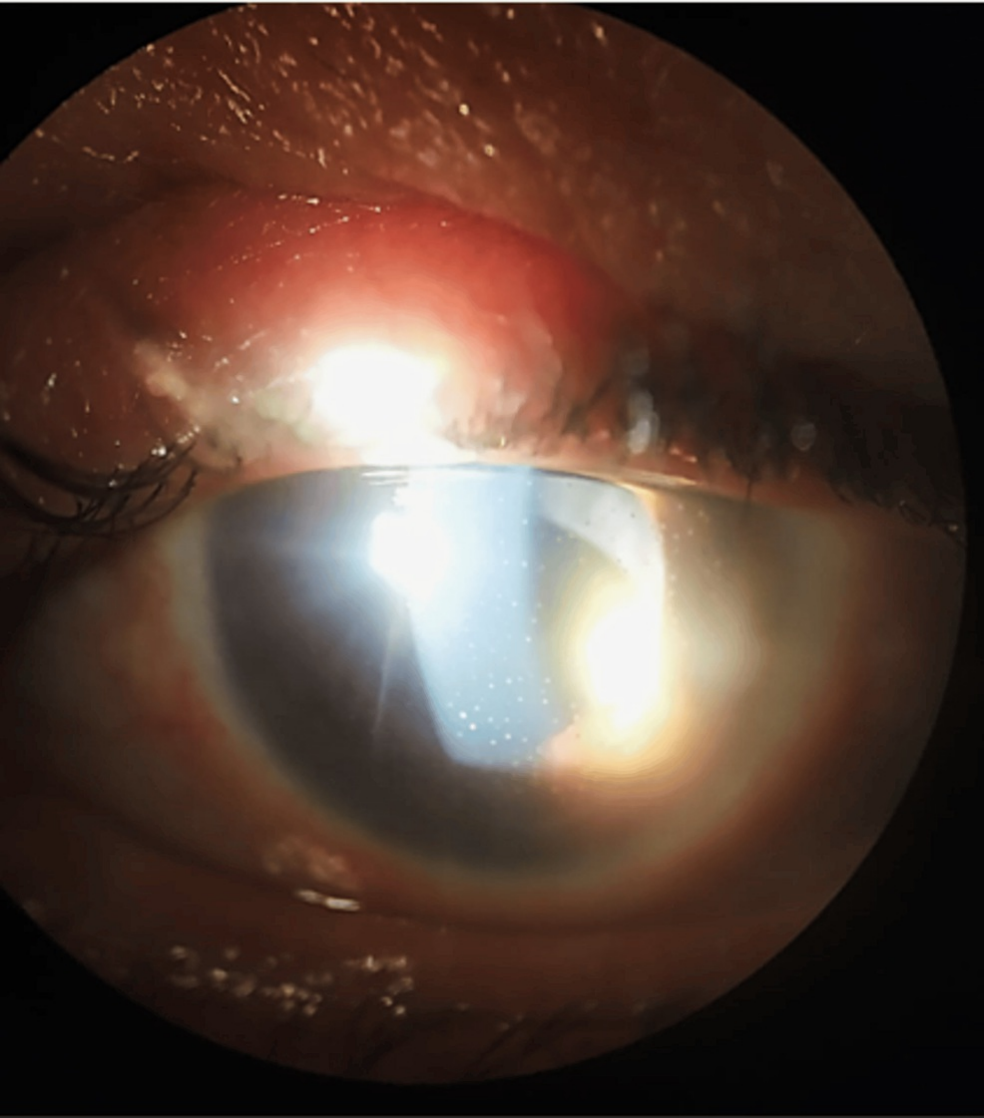
Slit lamp photograph of the left eye showing diffuse medium-sized keratic precipitates

During the follow-up period, two months after surgery, the patient's left eye IOP gradually increased, and diffuse medium-sized keratic precipitates were observed (Figure 1). A gonioscopy revealed a synechial closed angle in the left eye. Suspecting an underlying cause for the recurrent hypertensive anterior uveitis, an aqueous humor sampling was performed, and polymerase chain reaction (PCR) testing detected the presence of CMV-DNA (25675.18 copies/mL of DNA). Consequently, the patient was started on topical ganciclovir 0.15% four times a day and oral valaciclovir 1 g twice daily, along with topical prednisolone (dexamethasone phosphate 0.1% every three hours) and IOP-lowering medication (timolol 0.5% -dorzolamide 2% fixed combination twice a day and brimonidine 0.15% twice a day).

Over the subsequent follow-up visits, the patient's left eye IOP and ocular inflammation were closely monitored. The combined therapeutic approach resulted in a favorable response, achieving controlled IOP and the resolution of anterior uveitis. Upon the last visit, her final BCVA was 20/20 in the right eye and 20/60 in the left eye, with stabilized IOP at 12 mmHg in both eyes. Additionally, her hematological study returned negative results for infectious diseases, including HIV.

## Discussion

Hypertensive anterior uveitis represents a challenging clinical scenario characterized by elevated IOP and concurrent ocular inflammation. This case report highlights the diagnostic journey of a 66-year-old immunocompetent female presenting with refractory hypertensive anterior uveitis, ultimately identified as being caused by CMV infection.

The patient's prior history of acute angle closure glaucoma could have predisposed her to the development of a hypertensive crisis. Topiramate, which she had been taking, is also associated with ocular side effects, including secondary angle-closure glaucoma [6]. Discontinuation of topiramate and the prompt initiation of hypotensive medication effectively managed the initial hypertensive crisis. However, the recurrence suggests an unlikely association with the previous episode.

Bilateral phacoemulsification was performed because of primary angle closure, which initially resulted in a normalized IOP without the need for hypotensive medication. However, the recurrence of elevated IOP and the development of keratic precipitates necessitated further investigation.

The aqueous humor sampling and PCR testing were vital in diagnosing CMV-induced hypertensive anterior uveitis. As a member of the herpesvirus family, CMV can cause ocular complications, including anterior uveitis, endotheliitis, and glaucoma, even in immunocompetent individuals [2-5]. Aqueous humor analysis enables a swift and accurate clinical diagnosis, with the detection of CMV-DNA confirming the virus's direct involvement in the pathogenesis of the patient's uveitis [5].

Prompt initiation of antiviral treatment targeting CMV (oral valaciclovir), along with the concurrent use of topical corticosteroids and IOP-lowering medications, successfully managed the patient's ocular condition, as reported before [7]. The collaborative efforts of ophthalmology and infectious disease specialists played a pivotal role in the successful management of this complex case.

## Conclusions

This case report highlights the importance of considering CMV as a possible etiology in cases of refractory hypertensive anterior uveitis. The patient's history of angle closure glaucoma and topiramate use added complexity to the diagnostic process. Aqueous humor sampling and PCR testing were crucial in diagnosing CMV-induced uveitis. Prompt initiation of oral valaciclovir resulted in a favorable response.

This case emphasizes the need for increased awareness of CMV in refractory cases and the need for a comprehensive approach to optimize patient care.

## References

[1] Jap A, Chee SP (2011). Viral anterior uveitis. Curr Opin Ophthalmol.

[2] Chee SP, Jap A (2008). Presumed fuchs heterochromic iridocyclitis and Posner-Schlossman syndrome: comparison of cytomegalovirus-positive and negative eyes. Am J Ophthalmol.

[3] Chan NS, Chee SP, Caspers L, Bodaghi B (2018). Clinical features of CMV-associated anterior uveitis. Ocul Immunol Inflamm.

[4] Chee SP, Bacsal K, Jap A, Se-Thoe SY, Cheng CL, Tan BH (2008). Clinical features of cytomegalovirus anterior uveitis in immunocompetent patients. Am J Ophthalmol.

[5] Zhang J, Kamoi K, Zong Y, Yang M, Ohno-Matsui K (2023). Cytomegalovirus anterior uveitis: Clinical manifestations, diagnosis, treatment, and immunological mechanisms. Viruses.

[6] Abtahi MA, Abtahi SH, Fazel F, Roomizadeh P, Etemadifar M, Jenab K, Akbari M (2012). Topiramate and the vision: a systematic review. Clin Ophthalmol.

[7] Sira M, Murray PI (2007). Treatment of cytomegalovirus anterior uveitis with oral valaciclovir. Ocul Immunol Inflamm.

